# Collection of the digital data from the neurological examination

**DOI:** 10.1038/s41746-025-01659-2

**Published:** 2025-05-01

**Authors:** Bruno Kusznir Vitturi, Patrik Theodor Nerdal, Walter Maetzler

**Affiliations:** Department of Neurology, University Hospital Schleswig-Holstein and Kiel University, Kiel, Germany

**Keywords:** Neurology, Neurological manifestations

## Abstract

This review presents the *status quo* of how far the digitalization of elements of the neurological examination has progressed. Our focus was on studies that assessed the examination conducted in person, rather than through telemedicine platforms. Five hundred and twenty studies were included in this systematic review. The digital tools covered ten elements of the neurological examination: gait (173, 33%), motor system (149, 29%), eyes (85, 16%), cognitive functions (53, 10%), sensory system (47, 9%), balance (35, 7%), other movements (24, 5%), other cranial nerves (24, 5%), coordination (10, 2%), and autonomic nervous system (9, 2%). Most of the tools were portable (442, 85%), and in 215 studies (41%) the devices were wearable. The cost of the digital tools used was described and discussed in 167 (32%) studies. Most devices (61%) had a low complexity, and half required high additional analytic effort.

## Introduction

New digital tools are constantly being introduced into clinical practice. This implementation is multifaceted and involves the use of digital technology in various stages of patient care, from electronic medical records to artificial intelligence for the diagnosis and evaluation of progression, treatment success, and even treatment^1,2^. Most clinical diagnoses are made based on information obtained from the doctor’s (first) personal contact with the patient, which almost always includes a physical examination^3,4^. In line with this, a large survey of UK doctors found that 70% said the physical examination was ‘almost always valuable’ in the hospital setting. However, in contrast to practically all complementary exams, such as imaging and biofluid analyses, the digitalization of the physical examination is practically not implemented in any medical discipline. Fortunately, the digitalization of the physical examination is subject of great interest in the medical-scientific community^5^ and doctors understand that this digitalization process could not only lead to easier conduction of the physical examination but also to more and better data^6^.

The digitalization of the neurological examination (NE) may have particularly high potential due to the following reasons. Firstly, NE contains many elements that have to do with movements of the body or parts of the body, as well as with the language and vocalizations of the person being examined. The recording of these aspects is very advanced in the field of digital technology and associated algorithms (optical camera systems, eye trackers, wearable devices detecting mobility aspects, and microphones can be mentioned here), and in many versions even already available in consumer technology. Secondly, many diseases are chronically progressive or relapsing, and the clinical manifestations often change only subtly during the disease or a relapse^7^. One strength of digital technology can be that even very subtle changes can be detected, but also that digital data can be stored and compared very accurately and objectively over time. This argument also applies, of course, to the evaluation of treatment response in the context of neurological diseases. Thirdly, the NE is complex and therefore difficult to learn and difficult to compare between different examiners. Standards for the various elements of the NE are largely lacking. It is interesting to note in this context that two-thirds of medical trainees reported they were never observed by a consultant when undertaking physical examination, and one-third reported that consultants have never demonstrated their use of the physical examination to them^7^. Digital technology can substantially facilitate the development of skills in doctors in training, and they can substantially facilitate the comparison between examiners.

As a neurological specialty, it could therefore make sense to advance the digitalization of the NE as efficiently and consensually as possible. To this end, it is necessary to present the *status quo* of how far the digitalization of elements of the neurological examination has progressed, which is the purpose of this systematic review.

## Results

**Fig. 1:**
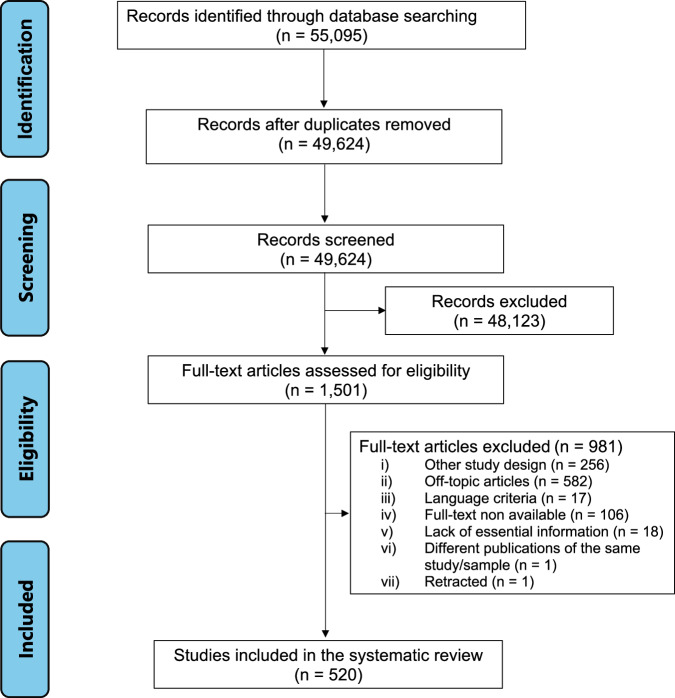
PRISMA flowchart.

The search terms led to 49,535 hits. After removing the duplicates, 49,414 articles were screened by reading the title and abstract. Finally, 520 articles met all the eligibility criteria, and their full texts were read (Fig. 1). The studies were carried out in 48 countries from 6 continents. Five countries accounted for more than 50% of the scientific publications: The United States of America (149, 28.6%), Germany (48, 9.2%), The United Kingdom (37, 7.1%), Italy (33, 6.3%), and Switzerland (28, 5.4%). Overall, 68,063 study participants were included. The median year of publication of the studies was 2018.

Four hundred and twenty-four studies (81.5%) investigated the use of digital devices on patients with neurological disorders, while 96 (18.5%) studies involved only healthy persons. In 234 studies (45.0%), there was a comparison between patients and controls. In 48 studies (9.2%), researchers included patients with more than one neurological disorder. Parkinson’s disease (140, 26.9%), Multiple Sclerosis (51, 9.8%), and Stroke (57, 11.0%) were the most investigated diseases. In 219 studies (42.1%), the authors intended to validate the digital method, and in 325 studies (62.5%) there was a comparison between the proposed new method and the current clinical gold standard assessment. Most of the technological devices were portable (442, 85.0%), and in 215 studies (41.3%), the devices were wearable. The cost of the devices used was described and discussed in 167 (32.1%) studies. Fifty-six (10.8%), 146 (28.1%), and 318 devices (61.1%) were classified as high, intermediate, and low complexity, respectively. One hundred and eighteen (22.7%), 143 (27.5%), and 259 (49.8%) devices generated immediate, intermediate, and lengthy results, respectively.

Most of the elements of the traditional NE have already been explored with digital devices. This includes (in descending order) gait (173 studies, 33.3%), motor system (149 studies, 28.6%), eyes (85 studies, 16.3%), cognitive functions (53 studies, 10.2%), sensory system (47 studies, 9.0%), balance (35 studies, 6.7%), other movements (24 studies, 4.6%), other cranial nerves (24 studies, 4.6%), coordination (10 studies, 1.9%), and autonomic nervous system (9 studies, 1.7%). In 60 studies (11.5%), aspects from more than one element were investigated. Table 1 presents more detailed information, including pathologies typically evaluated within the NE that have been addressed with these digital devices.

**Table 1:** Elements of the neurological exam and neurological signs observed with digital devices

Element of NE	Neurological sign
Gait	Gait abnormalities
Motor system	
Muscle strength	Paresis/plegia
Muscle tonus	Spasticity/hypertonia
Involuntary movements	Tremor, dyskinesia, chorea
Deep Tendon Reflexes	Hyperreflexia/hyporreflexia
Eyes	
1) Visual field	Hemianopsia, quadrantopsia
2) Visual acuity	Low visual acuity, amaurosis
3) Eye fundus exam	Papilledema
4) Extraocular motility and alignment/eye movements	Saccades, nystagmus
5) Vestibulo–ocular reflex	Abnormal reflex response
6) Pupillary size	Myosis and mydriasis
7) Pupillary light reflex	Abnormal reflex response
8) Blink reflex	Abnormal reflex response
Sensory system	
Proprioception	Hypoesthesia
Thermal sensitivity	Hypoesthesia
Pallesthesia	Hypoesthesia
Pain sensitivity	Hypoesthesia
Cognitive functions	Cognitive impairment and altered level of consciousness
Balance	Balance abnormalities
Other movement assessments	Rigidity, hypokinesia, bradykinesia
Other cranial nerves	
Facial movements	Facial paralysis
Voice and speech	Dysphonia and Dysarthria
Swallowing	Dysphagia
Autonomic Nervous System	Baroreflex, oculocardiac reflex, sweating abnormalities
Coordination	Dysdiadochokinesia, dysmetria, ataxia

In the cohorts studied here, digital technology was most often used to investigate aspects of gait, and the Inertial Measurement Unit (IMU) was the most frequently used instrument. In general, the complexity was variable, but the time taken to analyze and process the data was most often substantial. One hundred and fifty-seven (90.7%) and 119 (68.8%) of the devices were portable and wearable, respectively (Table 2). The associated costs were reported in 54 (31.2%) studies. Many studies suggest that the digital examination of this element can provide important additional information on (early) detection, progression, and treatment response in various neurological diseases. For example, gait asymmetry and bilateral coordination during straight walking have been linked to prognosis and functional outcomes in multiple sclerosis (MS)^8^. Several posturography parameters related to static balance have been associated with prodromal Huntington’s disease (HD)^9^ or were used to objectify chronic subjective dizziness^10^. In CACNA1A disorders, characterized by mutations in the calcium voltage-gated channel subunit alpha1 A, gait parameters such as gait speed, step width, and ankle range of motion have shown abnormalities, even when gait was clinically judged as unaffected by the investigator^11^. Additionally, a significant number of variables derived from inertial movement units have been associated with disease progression in Parkinson’s disease (PD)^12,13^, and specific gait parameters have been linked to functional improvement after acute stroke^14^.

**Table 2:** Detailed characterization of digital devices described in the literature

	Gait	Motor system	Eyes	Sensory system	Cognitive functions	Balance	Other movements	Other cranial nerves	Autonomic nervous system	Coordination
Complexity										
High	16 (9.2%)	24 (16.1%)	5 (5.9%)	13 (27.6%)	9 (17.0%)	3 (8.6%)	0 (0.0%)	2 (8.3%)	1 (11.1%)	2 (20.0%)
Intermediate	71 (41.0%)	51 (34.2%)	10 (11.8%)	7 (14.9%)	4 (7.5%)	11 (31.4%)	5 (20.8%)	6 (25.0%)	5 (55.5%)	3 (30.0%)
Low	86 (49.7%)	74 (49.7%)	70 (82.3%)	27 (57.4%)	40 (75.5%)	21 (60.0%)	19 (79.2%)	16 (66.7%)	3 (33.3%)	5 (50.0%)
Time for analysis										
Immediate	9 (5.2%)	21(14.1%)	38 (44.7%)	26 (55.3%)	26 (49.0%)	7 (20.0%)	9 (37.5%)	2 (8.3%)	3 (33.3%)	0 (0.0%)
Intermediate	33 (19.1%)	44 (29.5%)	26 (30.6%)	10 (21.3%)	18 (34.0%)	11 (31.4%)	8 (33.3%)	6 (25.0%)	3 (33.3%)	4 (40.0%)
Lengthy	131 (75.7%)	84 (56.4%)	21 (24.7%)	11 (23.4%)	9 (17.0%)	17 (48.6%)	7 (29.2%)	16 (66.7%)	3 (33.3%)	6 (60.0%)
Portable	157 (90.7%)	115 (77.2%)	76 (89.4%)	31 (66.0%)	46 (86.1%)	27 (77.1%)	24 (100.0%)	22 (91.7%)	6 (66.6%)	7 (70.0%)
Wearable	119 (68.8%)	74 (49.7%)	14 (16.5%)	13 (27.6%)	7 (13.2%)	18 (51.4%)	6 (25.0%)	4 (16.7%)	2 (22.2%)	5 (50.0%)
Cost	54 (31.2%)	43 (28.8%)	25 (29.4%)	13 (27.6%)	22 (41.5%)	11 (31.4%)	8 (33.3%)	9 (37.5%)	2 (22.2%)	3 (30.0%)
Clinical validation	74 (42.8%)	64 (43.0%)	20 (23.5%)	24 (51.1%)	26 (49.0%)	16 (45.7%)	13 (54.2%)	13 (54.2%)	1 (11.1%)	4 (40.0%)

Although there are good examples demonstrating the usefulness of digital gait assessment, the literature also includes evidence that highlights concerns regarding the widespread digitalization of this element of the NE. Hickey et al. showed that there may be a poor agreement between two different digital ways of assessing gait variability and asymmetry^15^. A Dutch group found that the assessment of 5 meters of gait with a single body-fixed sensor had a high sensitivity but a low specificity in detecting Parkinsonian gait^16^. In parallel, Hansen et al. demonstrated that temporal gait parameters showed only poor to moderate reliability in a cohort of neurogeriatric patients^17^. In other cases, the results of gait analyses were accurate and reliable, but they were not tested on patients with neurological diseases, which undermines their potential for incorporation into clinical practice^18–20^. Indeed, when gait assessment technology is applied to patients and not just healthy individuals, there is often a significant loss of accuracy^21^. In extreme cases, digital gait analysis has been shown not to be a valid method or to offer no real added value compared to clinical examination^22,23^. Dewey et al. found that the baseline gait and balance measures failed to predict future rates of symptom progression in PD^24^.

### Motor system

Global motor function has been investigated in most of the literature on digital examinations of the motor system. The most common type of device used was IMU. The complexity was variable, but the time to obtain clinically relevant results was long in most studies. More than three-quarters (77.2%) of the devices were portable, and 74 (49.7%) were wearable. The cost was reported in 43 (28.8%) studies. In one study a portable system was developed that assessed patients with cerebellar ataxia through the instrumented versions of 9 commonly used neurological tests^25^. The authors found a performance accuracy of 97%. Another study suggested that the concept of transitory ischemic attack (TIA) could be redefined with an exoskeleton that detected and quantified specific patterns of abnormal upper limb motor behavior after a TIA in subjects considered “asymptomatic”^26^. Likewise, a further study used wearable sensors to determine the activity (lying, sitting, standing, walking) of individuals at risk for HD and were successful in identifying specific patterns associated with the prodromal phase of the disease^27^. In an American study, the researchers strengthened the prognostic value of the motor assessment using a robotic measure that were associated with stroke outcomes^28^.

Other studies were dedicated to assessing specific parts of the motor exam such as involuntary movements, muscle tone, muscle strength, and deep tendon reflexes. One study showed that the results of the quantitative assessment of tremor and rigidity in people with PD provide a tailor-made treatment of the disorder^29^. An Italian study provided solid evidence that the tremor stability index obtained by analyzing data from a camera has a specificity and accuracy of 95% and 92% in differentiating Parkinsonian tremor from essential tremor, respectively^30^. Another study suggested that it is possible to track the close progression of tropical spastic paraparesis with the digital assessment of spasticity^31^. Interestingly, IMUs may be useful to assess spasticity among comatose patients^32^. Only three studies were devoted to quantifying the deep tendon reflexes, but all had positive results^33–35^.

### Eyes

Eight aspects of the NE of the eyes were studied with digital tools: visual field, visual acuity, extraocular motility and alignment/eye movements, vestibulo-ocular reflex, pupillary size, pupillary light reflex, and blink reflex. The eye tracker was the most used tool for this purpose. Overall, most of the tools were not complex but the time it took to obtain results was variable. Seventy-six (89.4%) devices were portable, and 14 (16.5%) were wearable. The cost was reported in 25 (29.4%) studies. One study used a low-cost eye tracker that provided more accurate results in the assessment of optokinetic nystagmus eye movement in comparison with the clinical examination^36^. Another study validated an easy-to-use device made up of cameras and video processing algorithms that were useful for identifying visual pursuit in comatose patients, a clinical context in which traditional NE has inherent limitations^37^. A German group used an eye tracker to compare saccade and pupil abnormalities in patients with manifest alpha-synucleinopathies. They found this technology potentially useful to diagnose the prodromal stage of the diseases^38^.

Not all the studies that have used the eye tracker have found positive results that can recommend its use in clinical practice. One study compared the eye tracking performance and other common concussion evaluations and found that the technology is not superior to the traditional clinical evaluation of patients with a concussion^39^. Similarly, the digital assessment of saccadic eye movements revealed that there are significant technical limitations that may prevent its adoption by clinicians^40^. In another study, a head-mounted saccadometer provided a quantitative assessment of saccadic eye movements but the results did not have any clinical significance^41^.

A camera and deep-learning system were used to diagnose papilledema. The system discriminated disks with papilledema from normal disks and disks with papilledema abnormalities with an AUC of 0.99^42^. Several studies addressed the pupils’ size and the pupillary light reflex with pupillometers. The results are consistent, and the quantitative data generated are highly associated with the prognosis of patients in the neurocritical care unit^43–46^. Regarding the assessment of the vestibular-ocular reflex (VOR) complex and expensive eye tracking systems are used to detect pathologies that are difficult to see with the naked eye^37^, However, there are also indications that cheaper digital devices such as smartphones are comparable^46^. Although the devices used can detect a vestibular failure,in most cases, digital assessment of the VOR was unable to distinguish stroke from peripheral vestibular loss in patients with acute vestibular syndrome^47–50^. Conflicting evidence also exists in the case of the accuracy and benefit of digital assessment of visual acuity. It was used exclusively to assess the visual acuity of MS patients^51,52^. Few studies aimed to assess the visual field and the results do not favor digital assessment over manual examination^53,54^. Finally, there was only one study that specifically assessed the blink reflex digitally. The authors developed a high-speed videography-based device that provided a fast, objective, and quantitative metrics of the blink reflex^55^.

### Cognitive functions

The most common technologies used to assess cognitive function were eye trackers, tablets, and computers. Most of these devices, capable of assessing various cognitive functions, were of low complexity and required no additional time to generate valuable results. Forty-six (86.1%) devices were portable, and 7 (13.2%) were wearable. Cost was discussed in 22 (41.5%) studies. In one study, a computerized assessment battery for cognition was developed that were useful to detect mild cognitive impairment and dementia^56^. Another study used an eye tracker to investigate visual short-term memory through eye movements in the context of preclinical familial Alzheimer’s Disease^57^. In parallel, a Swiss group showed that eye tracking in puzzle games can act as a supplemental source of data for the assessment of cognitive performance^58^. Digital biomarkers demonstrated to predict the prognosis of people at risk for dementia within three years^59^. Moreover, a computer software was found to aid the diagnosis of dementia by quantifying and systematically evaluating differences in expressing facial emotions among people with cognitive impairment^60^. In another case, the digital assessment generated information that could not be inferred from the conventional neurological cognitive tests. The authors administered a digital version of the Digit Symbol Substitution Test and identified previously unrecognized patterns of ‘writing’ and ‘thinking’ time that were associated with cognitive function^61^.

### Sensory system

Sensory exams that implemented digital tools measured temperature, pain, vibration, position sense, and tactile stimulation. A huge variety of devices have been described, and most have low complexity and generate results immediately. Almost two-thirds (66.0%) of the devices were portable. Thirteen (27.6%) were wearable, and 13 (27.6%) had their cost mentioned. Patients with diabetic neuropathy were the most often investigated population. One study describes a portable quantitative sensory testing device that gives scores for vibration perception threshold, cold perception threshold, and warm perception threshold. It was useful to screen peripheral neuropathy among patients with diabetes^62^. A Chinese group used a digital vibration sensory tester that was useful for diagnosing early peripheral neuropathy in workers exposed to neurotoxic agents^63^. A further study developed an exoskeleton that quantified prevalence and severity of kinesthetic deficits of the upper limb poststroke with greater accuracy^64^.

Also excessively complex digital tools and tools producing negative results were published, making them unlikely to be included in clinical practice. For example, a complex robot-based assessment of the proprioceptive function failed to be superior to the traditional exam in predicting the prognosis of stroke patients^65^. Another study found that the digital assessment of pallesthesia and thermal sensitivity was not useful for the subclinical diagnosis of diabetic peripheral neuropathy^66^. Moreover, the robotic assessment of finger proprioception in MS patients revealed to have only good reliability^67^.

### Balance

The most commonly used digital devices for the assessment of balance were boards, cameras, and accelerometers. Most technologies had a low or intermediate complexity. Twenty-seven tools (77.1%) were portable, and 18 (51.4%) were wearable. Eleven (31.4%) studies discussed the associated costs. One study found that accelerometer-based metrics of sway may be a more sensitive method of diagnosing PD progression than the UPDRS scores^68^. The digital assessment may be at least equivalent to the traditional evaluation. A Chinese group developed an intelligent system based on a camera and machine learning that had an intraclass correlation coefficient of 0.94 in detecting postural characteristics of PD patients^69^. The quantitative assessment of balance demonstrated to predict the probability of independent gait after a stroke^70^. In another study, a balance assessment software incorporated into a common video game device proved to be a reliable method of assessing balance as an example of a cheap, portable, and convenient method^71^.

### Other movement assessments

Technology was used to assess and identify bradykinesia, akinesia, rigidity, micrography, and hypomimia. The literature describes various types of digital devices used to assess this element, with cameras, tablets, smartphones, computers, and IMUs being the most utilized^72–76^. Overall, the complexity was low. The time to get clinically meaningful data was highly variable. All the devices were portable, and 6 (25.0%) were wearable. Cost was mentioned in 8 (33.3%) studies. A tablet-based app demonstrated to have higher reliability and responsiveness in capturing bradykinesia-related tasks than did clinician ratings^77^. In a Japanese study, the researchers quantitatively assessed the finger-tapping test of PD patients and found an association between the contact force in a touch sensor and the UPDRS score^78^. Similarly, a Swedish study classified PD patients estimating speed, amplitude, rhythm, and fatigue of the tapping symptoms obtained from a camera with an accuracy of 88%^79^. Two studies used a 3D-ultrasound device and an IMU, respectively, to quantify and determine signs of bradykinesia to discriminate PD patients from controls^80,81^. One study reported the success of a self-administered test performed through a tablet-based application in quantifying bradykinesia of PD patients^82^. In a British study, the authors presented a new computer vision technology capable of assessing bradykinesia from common smartphone videos of finger-tapping test^77^.

### Other cranial nerves

Facial movement, swallowing and voice, and speech were investigated with digital tools. Video cameras and voice recorders were the most frequent type of device used. Most of the devices were of low complexity, and most of them required much time to obtain results. The vast majority (91.7%) were portable, and 4 (16.7%) devices were wearable. The cost was reported in 9 (37.5%) studies. One study demonstrated that patterns of facial weakness detected with facial recognition software were useful to distinguish patients with Myasthenia Gravis from controls^81^. A British study demonstrated that computer-assisted photography is superior to a manual method of monitoring of facial motor function^82^. Another study was successful in assessing this symptom using acoustic analysis of sounds recorded by a microphone^83–85^. Similarly, other studies used a voice recorder to assess the voice and speech and detect Alzheimer’s Disease, PD, and Amyotrophic Lateral Sclerosis^86–88^.

Few studies used digital devices to assess the cerebellar function and diagnose ataxia, dysmetria, and diadochokinesia, and they used different technologies. No device was simple to use, and the time taken to obtain the results was highly variable. Seven tools (70.0%) were portable, and 5 (20.8%) were wearable. One study used IMUs to assess the body symmetry of upper and lower limbs quantitatively to discriminate MS from healthy individuals^89^. Another study used an ultrasound-based recording device to accurately assess the joint angles during diadochokinetic movements^90^. A further study developed a complex mechatronic myohaptic unit to extract a common electrophysiological signature of a cerebellar deficit^91^.

Some studies aimed to use digital tools to diagnose signs of dysautonomia, and different types of instruments were used in this endeavor. Each study used a different technology. Only one (11.1%) device was highly complex, and 3 (33.3%) required a long time to obtain the results. Two-thirds were portable, and 2 (22.2%) were wearable. Cost was mentioned in 2 (22.2%) studies. A group of neurologists and technicians reported the development of an easy-to-use instrument that integrates physiological information from different already existing commercial modules to evaluate the autonomic nervous system in a standardized way^92^. Another study explored a neurological feature not commonly investigated by traditional physical examination and quantitatively assessed sudomotor function of people with PD to diagnose signs of dysautonomia^93^. Using a monitor of hemodynamic parameters, a further study found a significant correlation between orthostatic hypotension and decreased baroreflex sensory system in people with PD^94^. In one study, the authors did not find a relevant association between the results of a device designed to assess the baroreflex and standard autonomic tests^95^.

## Discussion

The review shows that the literature is vast regarding the use of digital devices in the assessment of elements of the NE. Results suggest that the DNE can bring numerous advantages over the conventional NE. Some of the digital devices used have proven to be highly accurate and capable of detecting neurological alterations more assertively than does conventional NE. Moreover, technology has also proven useful in monitoring patients with chronic neurological disorders more closely and predicting prognosis more accurately, compared to conventional NE. Many studies used digital devices successfully regarding feasibility, usability, validity, clinical efficacy, including detection of disease, measurement of disease course, and treatment response, to assess human neurological performance. All these characteristics indicate that such digital technology may have the potential to enable early diagnosis of neurological diseases, prevent medical errors, and guide clinicians towards more assertive and effective prevention and treatment strategies.

The literature also provides good examples of relatively simple devices that are increasingly being used, such as pupillometers^38,43–46^. We understand that ease of use is an essential and indispensable feature for the implementation of digital tools in the daily practice of neurologists. Future clinical studies should prioritize such devices, as many digital devices are still extremely complex and require significant effort and time to generate clinically relevant data. Moreover, many devices, such as robotic exoskeletons, require complex and time-consuming data analysis approaches that require the support of engineers and data scientists^26^, making their implementation in clinical practice unlikely.

The literature is uneven in some aspects. Scientific production is concentrated in a few countries and the use of digital devices is investigated in only a few neurological diseases. Furthermore, some articles fail to prove the real benefit of the digital assessment of neurological features. This result may be associated with the observation that not all studies have adequately validated the medical device or compared it with the current gold standard of NE.

Our review also highlights that some aspects of the neurological examination are more easily explored with digital devices than others. For example, the analysis of reflexes and meningeal signs, which are critical during the neurological exam, remains largely unexplored. In contrast, gait analysis has been the focus of most attempts to digitalize the neurological examination. However, it is still unclear how neurologists can standardize and summarize a digital protocol for gait assessment.

Also, the cost of digital evaluation is underreported. The cost of any new technology is a crucial point in supporting the decision to integrate it into clinical practice^96^. Traditional neurological examinations are low-cost, as neurologists rely on basic tools such as a tongue depressor, reflex hammer, and tuning fork. The digitalization of neurological examinations may be costly and may not be economically sustainable when considering the initial acquisition and development costs, along with the ongoing maintenance expenses. However, it should be kept in mind that the DNE can also lead to cost savings over time. Streamlined workflows, reduced film and storage expenses, and improved resource utilization contribute to long-term cost-efficiency^97^. Considering that NE is currently extremely complex and requires adequate training, the creation of an evidence-based DNE could reduce costs related to medical errors and unnecessary complementary exams. Moreover, a DNE can paradoxically be useful for countries in development with a lack of qualified doctors, as the training for using a new technological resource may be simpler compared to all the process of training specialized and qualified medical professionals. Studies investigating this aspect should be particularly encouraged.

This systematic review also shows that there are numerous research gaps in the field of DNE. Advancements in technologies that contribute to NE bring significant ethical concerns, particularly regarding data privacy, informed consent, and clinician accountability^98^. Given the sensitive nature of neurological data, it is crucial to implement strong data protection protocols. Moreover, patients must be thoroughly informed about how their data will be gathered, stored, and utilized, ensuring transparency and voluntary consent in accordance with ethical guidelines. Neurologists who use digital tools must also address potential liability issues, as reliance on these technologies could impact the accuracy of diagnoses and clinical decisions. This introduces the risk of errors if the tools malfunction or if their results are misinterpreted. As such, it is vital that clinicians maintain responsibility for the outcomes of clinical assessments, using digital tools as supportive aids rather than substitutes for professional judgment. Ethical standards must evolve continually to meet these challenges, ensuring that technological advancements are applied in ways that prioritize patient rights and uphold the integrity of medical practice^99^.

Few studies have explored the DNE from the doctor’s perspective. In some cases, the integration of digital devices into clinical practice may necessitate the involvement of an additional professional between the patient and clinician: a technology expert. Certain technologies require advanced, specialized training to collect, analyze, and interpret the results^100,101^. In this context, it is essential to assess how feasible and realistic it is to implement such devices in clinical settings. Furthermore, it is important to consider whether doctors would truly feel comfortable incorporating these tools into their practice, as it could potentially redefine the physician’s role in the medical consultation.

Moreover, although a substantial number of articles have already been published on the topic, we found no studies that reported efforts to unify the various elements of the NE into a single, comprehensive DNE. This represents a significant gap in the current literature, and we believe that the development of such an integrated approach will be a crucial area for future research. Unifying these elements could help streamline the digitalization process and enhance the clinical utility of DNEs, potentially improving both diagnostic accuracy and patient care.

Although this review is as comprehensive as possible, it has limitations. For example, we did not assess the quality of the original articles included in the analysis. In addition, the inclusion criteria and search strategy for scoping reviews, as was the case for this review, may be less stringent than for other systematic reviews, leading to subjectivity in the selection process. However, we have attempted to define all search terms and extracted variables as precisely as possible to provide a useful basis for future, more specific literature analyses that could happen, for example, at element level. Moreover, we excluded telemedicine aspects and studies on remote neurological examination to focus solely on the digital aspect that could be added to the “pure clinical” neurological examination. Our goal was to assess their diagnostic capabilities without the complexity of telecommunication infrastructure.

The digitalization of the neurological examination has significant potential to increase diagnostic accuracy, monitor disease progression and improve patient care by providing objective, reproducible measurements. This review shows that many elements of the neurological examination, particularly gait and motor function, are already being effectively assessed with digital tools. Although challenges remain, including device complexity and time requirements, continued development of user-friendly and efficient technologies promises to streamline implementation in clinical practice. Future research should focus on validating these tools across a wider range of neurological conditions and simplifying their integration to benefit both clinicians and patients. As the field evolves, digital tools may become an invaluable asset in providing our patients with an accurate neurological examination.

## Methods

This systematic review adheres to the guidelines established by Arksey and O’Mally^101^, the recommendations provided by the Joanna Briggs Institute^102^, and the Preferred Reporting Items for Systematic Reviews and Meta-Analyses for scoping reviews (PRISMA-ScR)^103^ (Supplementary material).

### Research question and search strategy

The main research question of this review was “How far has the digitalization of elements of the neurological examination progressed?”. The question was framed following the PCC (population, concept, and context) principle^104^. We systematically searched PubMed/MEDLINE from 1 August 2023 to 01 August 2024 using the keywords as presented in the Table 3. No time limits were set for the publication date. Manuscripts written in English, German, Italian, Spanish, French, or Portuguese were exported into Zotero (v. 6.0.30, Virginia, USA), and abstracts were read. They were selected for the next step of the search if they investigated at least one element of the NE with at least one digital tool in healthy adults and/or adult people with a neurological condition. The NE components sought were based on the structure outlined in a classic textbook of neurological semiology^105^, which is widely regarded as an authoritative and comprehensive reference for both clinicians and students. Subsequently, we obtained the full text of the remaining articles, re-evaluated their eligibility, and determined their ultimate inclusion or exclusion.

**Table 3:** Detailed search strategy

PubMed/MEDLINE	(“DIGITAL” OR “DEVICE” OR TECHNOLOG* OR “APP” OR “SENSOR” OR “ARTIFICIAL INTELLIGENCE”) AND (EXAM* OR ASSESS* OR DETECT* OR MEASURE*) AND (NEUROLOG*).

Publications configured as reviews, letters to the editor, expert opinions, commentaries, conference proceedings, and editorials, and retracted papers were excluded. If the full text could not be found, or if an article had missing or uncertain key data, the corresponding author was contacted by e-mail up to two times, and the study was excluded if contact was unsuccessful. Articles were also excluded if they utilized digital tools for remote or telemedicine-based neurological examinations or if their primary aim was to promote neurological rehabilitation. In the case of publications referring to the same research sample and using the same technology to assess the same neurological feature, we only included the article with the largest sample.

### Data extraction

We extracted the following essential information from the included studies: authors, title, year of publication, country, and number of participants. We also recorded whether the studies involved participants with neurological diseases and/or healthy individuals. For the digital analysis of the NE, we documented the type of device responsible for extracting the information and/or containing the sensor (e.g., tablet, smartphone), identified the specific elements of the NE being investigated, and noted the clinical neurological signs that could potentially be digitally detected. Additionally, we gathered details on the characteristics of the digital assessment, including (i) the complexity of the device, (ii) the time required to obtain results, (iii) whether the study attempted to clinically validate the proposed device, (iv) the portability of the device, (v) the cost of the assessment, and (vi) its wearability. Detailed definitions of the terms and classification strategies are provided in the Table 4.

**Table 4:** Classification of the characteristics of the devices of interest

Complexity of the device	The complexity of the instruments was classified as high, intermediate, or low. The complexity analysis was based on the difficulty of assessing neurological function based on homologous neurological tests already carried out in clinical practice. For example, an assessment which requires the use of a complex structure (e.g., a robotic exoskeleton), following complex specific instructions, and that is time-consuming to perform the analysis was classified as high complexity. In contrast, an instrument that does not depend on specific instructions and can be used by doctors without any prior training or information was classified as low complexity.
Time required to obtain results	The time taken to obtain the clinically meaningful results was categorized as time-consuming, intermediate, and immediate. Instruments that required the interpretation of the raw information by a non-medical professional and took more than 1 day to interpret the results were classified as “time-consuming”. Devices that provided results with potential value for the clinic immediately after the assessment and did not require any other professional to analyse the data were classified as “immediate”. Instruments whose data still required a second assessment but that could be obtained on the same day were classified as intermediate.
Aspect of the conventional neurological exam	The classification followed the summary description of the neurological examination that can be found in common textbooks of neurological semiology. The proposed digital assessment was also classified as “multidomain” if it involved the assessment of more than one domain of the traditional neurological examination (e.g., motricity and sensory system).
Ease with which a device can be carried or moved from one location to another	We defined portable devices as those that can be transported from one place to another easily and do not require specific and complex care when installing them in the new location, such as a tablet.
Cost of the assessment	We recorded whether the authors classified cost of the digital device along with the main results as low. If the authors did not specifically discuss or mention the cost, we did not infer it.
Wearability	The device was classified as wearable if it was designed specifically to be worn on the body

### Data evaluation and synthesis

First, we classified all studies according to their focus on the element(s) of the NE. Second, we summarized for every element of the NE the main results as presented in the data extraction paragraph above. Categorical data related to the six characteristics of the digital assessment described above were expressed as proportions. Some relevant studies were also described narratively, providing concrete examples of how technology was applied in each category of the NE.

## Supplementary information

Supplementary material

## Data Availability

Data is provided within the manuscript or supplementary information files. Additional data may be available upon request.
