# Author Correction: Collection of the digital data from the neurological examination

**DOI:** 10.1038/s41746-025-01819-4

**Published:** 2025-07-16

**Authors:** Bruno Kusznir Vitturi, Patrik Theodor Nerdal, Walter Maetzler

**Affiliations:** Department of Neurology, University Hospital Schleswig-Holstein and Kiel University, Kiel, Germany

**Keywords:** Neurology, Neurological manifestations

Correction to: *npj Digital Medicine* 10.1038/s41746-025-01659-2, published online 01 May 2025

In this article, the corresponding author was inadvertently designated only to “Bruno Kusznir Vitturi” but it should have been “Bruno Kusznir Vitturi” and “Walter Maetzler”. The original article has been corrected.

